# On Flexion of the Limbs as a Means of Suspending and Even Arresting Arterial Hæmorrhage

**Published:** 1852-08

**Authors:** 


From the New Orleans Med. and Surg. Journal.

*Of Flexion of the Limbs as a Means of Suspending and even Arresting Arterial Hæmorrhage.*

As arterial hæmorrhage is at all times more or less dangerous and alarming, it becomes proper for us to notice all the means best calculated to put a stop to the flow of blood proceeding from divided vessels. To this end, we are pleased to notice that Dr. Bobillier has turned his attention to this subject—the views of whom we shall abridge from the February number for 1852 of the *Journal des Connaissances Medico-Chirurgical.*

This gentleman has found, from experiment, that when certain arteries, situated about the joints of limbs, are wounded, the hæmorrhage therefrom may be arrested permanently, by flexing the limb forcibly upon itself. By this means he arrested a hæmorrhage from a wounded radial artery; and in another case, the same means succeeded after compression, etc., had been fairly tried and failed.

The third was the case of a man whose brachial artery was wounded by a blow with a knife, just in the bend of the arm, at the usual place of venesection—the hæmorrhage was frightful, and the patient was so situated, and the accident was so unexpected that the application of a ligature was utterly impracticable in the case. Violent and permanent flexion of the fore-arm upon the arm arrested the bleeding.

Dr. Bobillier deprecates any desire to place flexion of a limb in competition with the ligature, for arresting hæmorrhage. He contends, however, that it is a precious means, under certain circumstances—when the usual instruments for the application of ligatures are not at hand.

In 1834 M. Malgaigne, in his *Manuel de Medecine Operative*, speaks favorably of strong flexion of the fore-arm upon the arm, as a means of arresting hæmorrhage from wounds of the brachial artery. Four years thereafter, he mentions a case in which he arrested a hæmorrhage from the popliteal artery, by flexing the knees.

